# Efficacy of Rifampin Plus Clofazimine in a Murine Model of *Mycobacterium ulcerans* Disease

**DOI:** 10.1371/journal.pntd.0003823

**Published:** 2015-06-04

**Authors:** Paul J. Converse, Sandeep Tyagi, Yalan Xing, Si-Yang Li, Yoshito Kishi, John Adamson, Eric L. Nuermberger, Jacques H. Grosset

**Affiliations:** 1 Johns Hopkins University Center for Tuberculosis Research, Baltimore, Maryland, United States of America; 2 Department of Chemistry and Chemical Biology, Harvard University, Cambridge, Massachusetts, United States of America; 3 KwaZulu-Natal Research Institute for Tuberculosis and HIV, Nelson R. Mandela School of Medicine, University of KwaZulu-Natal, Durban, South Africa; Kwame Nkrumah University of Science and Technology (KNUST) School of Medical Sciences, GHANA

## Abstract

Treatment of Buruli ulcer, or *Mycobacterium ulcerans* disease, has shifted from surgical excision and skin grafting to antibiotic therapy usually with 8 weeks of daily rifampin (RIF) and streptomycin (STR). Although the results have been highly favorable, administration of STR requires intramuscular injection and carries the risk of side effects, such as hearing loss. Therefore, an all-oral, potentially less toxic, treatment regimen has been sought and encouraged by the World Health Organization. A combination of RIF plus clarithromycin (CLR) has been successful in patients first administered RIF+STR for 2 or 4 weeks. Based on evidence of efficacy of clofazimine (CFZ) in humans and mice with tuberculosis, we hypothesized that the combination of RIF+CFZ would be effective against *M*. *ulcerans* in the mouse footpad model of *M*. *ulcerans* disease because CFZ has similar MIC against *M*. *tuberculosis* and *M*. *ulcerans*. For comparison, mice were also treated with the gold standard of RIF+STR, the proposed RIF+CLR alternative regimen, or CFZ alone. Treatment was initiated after development of footpad swelling, when the bacterial burden was 4.64±0.14log_10_ CFU. At week 2 of treatment, the CFU counts had increased in untreated mice, remained essentially unchanged in mice treated with CFZ alone, decreased modestly with either RIF+CLR or RIF+CFZ, and decreased substantially with RIF+STR. At week 4, on the basis of footpad CFU counts, the combination regimens were ranked as follows: RIF+STR>RIF+CLR>RIF+CFZ. At weeks 6 and 8, none of the mice treated with these regimens had detectable CFU. Footpad swelling declined comparably with all of the combination regimens, as did the levels of detectable mycolactone A/B. In mice treated for only 6 weeks and followed up for 24 weeks, there were no relapses in RIF+STR treated mice, one (5%) relapse in RIF+CFZ-treated mice, but >50% in RIF+CLR treated mice. On the basis of these results, RIF+CFZ has potential as a continuation phase regimen for treatment of *M*. *ulcerans* disease.

## Author Summary

Buruli ulcer (BU) is caused by *Mycobacterium ulcerans* and its toxin, mycolactone. Since 2004, BU has been treated primarily with antibiotics rather than surgery and skin grafting. The current first-line regimen is an oral drug, rifampin (RIF), and an injectable drug, streptomycin (STR), daily for 8 weeks. Because STR injections are painful and have potential side effects, such as hearing loss, a replacement drug is sought. Emerging evidence of the efficacy of the anti-leprosy drug clofazimine (CFZ) against tuberculosis prompted an evaluation of CFZ + RIF as well as another all-oral regimen, RIF + clarithromycin (CLR) in a mouse model of BU. The results showed that RIF+CFZ initially acts more slowly against *M*. *ulcerans* than RIF+STR or RIF+CLR but it stops mycolactone production and is as good as RIF+STR and better than RIF+CLR at preventing relapse of infection. A drug regimen with a combination of three drugs, RIF+STR+CFZ, for one or two weeks followed by RIF+CFZ has the potential to limit the duration of STR treatment and achieve comparable cure.

## Introduction

Buruli ulcer (BU), or *Mycobacterium ulcerans* disease, has become a more readily treatable disease since 2004 with the transition from only surgery and skin grafting to antibiotic therapy with the combination of rifampin (RIF) and streptomycin (STR), the WHO preferred regimen or with RIF plus a fluoroquinolone or clarithromycin if STR is contraindicated, complemented by surgery in the case of extensive lesions to prevent or correct functional disabilities [1]. Although initial trials with the RIF+STR regimen included few reports of ototoxicity, objective measurements of hearing loss indicate that it may occur rather frequently [2]. For this reason and also operational issues with STR, an all-oral regimen is sought. Currently, there is hope that RIF plus clarithromycin (CLR) can be an acceptable oral regimen. However, this regimen would still require eight weeks of drug administration and may be complicated by gastrointestinal intolerance and the induction of CLR metabolism by RIF [3–7]. In addition, CLR is administered twice daily in some settings [5,6,8]. To date, a pilot study in Benin [9] has shown that patients with limited lesions can be successfully treated without relapse if treated with RIF+CLR for 8 weeks. Trials in Ghana have shown that treatment with RIF+STR for 4 weeks, followed by RIF+CLR for 4 weeks [10] or RIF+STR for 2 weeks, followed by RIF+CLR for 6 weeks [11] are as efficacious as RIF+STR for 8 weeks. In mice, RIF+CLR was inferior to RIF+STR and also inferior to the combination of the long-acting rifamycin, rifapentine plus CLR whether assessed by bactericidal activity, regression of footpad swelling, or recurrence (relapse) of swelling after treatment completion [12].

Unlike tuberculosis and leprosy, which have been treated with antibiotics for nearly 70 years, experience with successful antimicrobial treatment of BU has been limited to a decade. Alternative regimens and rhythms of treatment have had little opportunity for testing. Earlier studies of single drug treatment were uniformly disappointing, including a trial [13] of clofazimine (CFZ), a drug used in the combined drug regimen for multibacillary leprosy [14,15]. Based on recent favorable results in clinical [16] and mouse model studies [17,18], CFZ is receiving renewed attention for treatment of TB. Its mechanism of action is complex [19–22] and studies are ongoing to better define it. From murine model TB studies, it is apparent that its activity is delayed but features strong bactericidal and sterilizing attributes [17]. Because CFZ has never been studied in combination with other drugs in BU treatment, we hypothesized that together with RIF, CFZ could be a potent alternative to CLR and that a regimen of RIF+CFZ could be as effective as the standard RIF+STR regimen.

Here, we report that all three combinations, RIF+STR, RIF+CLR and RIF+CFZ achieved culture conversion by six weeks even against a high burden *M*. *ulcerans* infection in a mouse footpad model of BU. These regimens also stopped production of mycolactone A/B, the principal virulence factor of *M*. *ulcerans* [23]. CFZ had indeed poor initial activity when used as monotherapy but in combination with RIF, it was able to provide protection against relapse that was as effective as STR and more effective than CLR.

## Methods and Materials

### Bacteria

*M*. *ulcerans* 1059 (Mu1059), originally obtained from a patient in Ghana, and Mu1615, originally obtained from a patient in Malaysia, were generously provided by Dr. Pamela Small, University of Tennessee. Autoluminescent Mu1059 (Mu1059AL) was generated in our laboratory [24,25]. These strains all produce mycolactone A/B and this toxin kills macrophages and fibroblasts in vitro [26,27]. The Mu1617 type strain, originally isolated in Australia, has apparently lost the ability to produce mycolactone [26,28]. The Mu1059 strain was passaged in mouse footpads before use in these studies. The bacilli were harvested from swollen footpads at the grade 2 level, i.e., swelling with inflammation of the footpad [29].

### Ethics statement

All animal procedures were conducted according to relevant national and international guidelines. The study was conducted adhering to the Johns Hopkins University guidelines for animal husbandry and was approved by the Johns Hopkins Animal Care and Use Committee, protocol permit number MO11M103. The Johns Hopkins program is in compliance with the Animal Welfare Act regulations and Public Health Service Policy and also maintains accreditation of its program by the private Association for the Assessment and Accreditation of Laboratory Animal Care International.

### Antibiotics

RIF, CFZ, and STR were purchased from Sigma (St. Louis, MO). CLR was kindly provided by Abbott (Abbott Park, IL). STR and RIF were dissolved in distilled water, and CLR and CFZ were suspended in distilled water with 0.05% agarose. All drugs were given 5 days per week in 0.2 ml. RIF (10 mg/kg), CFZ (25 mg/kg), and CLR (100 mg/kg) were administered by gavage. STR (150 mg/kg) was administered by subcutaneous injection.

### Minimum Inhibitory Concentration (MIC)

of CFZ for *M*. *ulcerans* Mu1059, as well as for Mu1615, Mu1059AL, and Mu1617 was determined using a range of drug concentrations (0.06, 0.12, 0.25, 0.5, 1.0 and 2.0 μg/ml) by the proportion method on Middlebrook 7H11 medium. The MIC was defined as the lowest drug concentration preventing at least 99% of the growth observed on drug-free plates. The MIC for all strains tested was between 0.25 and 0.5 μg/ml.

### Infection and CFU analysis

BALB/c mice (N = 290), age 4–6 weeks (Charles River, Wilmington, MA), were inoculated in both hind footpads with approximately 3.3 log_10_ (2.0 x10^3^) CFU of Mu1059 in 0.03 ml PBS. Treatment was begun 7 weeks after infection when footpad swelling increased to approximately the grade 2 level. Treatment with RIF+STR, RIF+CLR, RIF+CFZ or CFZ alone was to be administered for 8 weeks (until week 15 after infection). Footpads were harvested before treatment initiation and then every two weeks from mice (5 footpads for CFU count, 3 for CFZ concentrations, 2 for ML detection) (Table 1). Mice were euthanized if they reached grade 3 swelling. Footpad tissue was harvested, minced with fine scissors, suspended in 1.0 ml PBS, serially diluted, and plated on Middlebrook selective 7H11 plates (Becton-Dickinson, Sparks, MD). Footpad homogenates from mice treated with CFZ were also plated in parallel on Middlebrook 7H11 containing 0.4% activated charcoal to reduce drug carryover effects. Plates were incubated at 32°C and colonies were counted after 12 weeks of incubation.

**Table 1:** Original experimental scheme to compare activities of RIF+STR, RIF+ CLR, RIF+CFZ and CFZ alone in *M*. *ulcerans*-infected footpads of BALB/c mice.

Drug regimens	Number of mice to sacrificed at the following time points							
	D-49	D 0	2 w	4 w	6 w	8 w	CFU (Swelling)	Total
**Controls**								
Untreated	5	5	5	5	5	5 (20)	30 (20)	50
RIF+STR			5 (10)	5 (10)	5 (10)	5 (10)	20 (40)	60
**Test**								
RIF+CLR			5 (10)	5 (10)	5 (10)	5 (10)	20 (40)	60
RIF+CFZ			5 (10)	5 (10)	5 (10)	5 (10)	20 (40)	60
CFZ only			5 (10)	5 (10)	5 (10)	5 (10)	20 (40)	60
**Total**	**5**	**5**	**25 (40)**	**25 (40)**	**25 (40)**	**25 (40)**	**115 (180)**	**290**

Due to animal welfare reasons, mice were sacrificed early as explained in text.

D, day; w, week; CFU, colony forming units; RIF, rifampin, 10mg/kg; STR, streptomycin, 150mg/kg; CLR, clarithromycin 100mg/kg; CFZ, clofazimine 25mg/kg; CLR and CFZ are given one hour after RIF to prevent adverse pharmacokinetic interactions [30].

### Analysis of mycolactone A/B

Footpads were harvested for detection of mycolactone by removal of soft tissue from the dorsal and ventral footpads and then immediately immersing it into a polypropylene Micrewtube tube with O-ring and screw cap (Simport Scientific, Beloeil, QC, Canada) containing 1500 μl absolute ethanol. Tubes were wrapped in foil and kept in the dark at room temperature. Samples were usually shipped overnight to the Kishi lab within 24 hours.

The extraction procedure has been described in detail previously [31]. The extracted material was spotted onto glass TLC plates along with synthetic mycolactone A/B standards and developed in 90:10:1 chloroform:methanol:water, air-dried, and dipped in boronic acid [17], heated for 5–10 seconds at 100°C. After wiping the glass back with acetone on a paper towel, the plate was placed on a UV lamp with a 365 nm filter. Fluorescent spot intensity was compared to that of the standards to estimate the amount of mycolactone A/B in the sample, as previously described [31].

### Pharmacokinetic analysis

For mice receiving CFZ alone or RIF+CFZ, footpad tissue from three of the five sacrificed mice at weeks 2, 4, 6 and 8 of treatment was submitted for quantification of CFZ. The footpad tissue was minced in 2 drops of PBS, placed in 900 μl absolute EtOH, and kept at 4°C until shipment to the Adamson lab. One hundred microliters of each prepared sample was added to 100 μl of water and 400 μl of acetonitrile, vortexed for 15 seconds and then centrifuged for 7 minutes at 16,000 × g at 4°C. After centrifugation 500 μL of the supernatant was combined with an equal volume of water in a capped LC/MS sample vial. LC/MS analysis was performed on an AB Sciex 5500 Q-Trap triple quadrupole MS system coupled with an Agilent 1200 system with refrigerated autosampler. A Waters Exterra 2.1 mm × 50 mm C18 column was used for chromatographic separation, along with an isocratic mobile phase consisting of 50% water and 50% acetonitrile, both solvents containing 0.1% (v/v) formic acid. The lower limit of quantitation was 0.048 μg per ml of tissue homogenate. The following parameters were used for concentration determination: for CFZ, transition 474/431.9; declustering potential 231 volts; collision energy CE 51 volts; entrance potential of 10 volts. LC/MS reagents, including LC/MS-grade acetonitrile, molecular grade formic acid and the clofazimine standard, were purchased from Sigma. Milli-Q water was used throughout the LC/MS procedure.

### Statistical analysis

GraphPad Prism 6 was used to compare group means by student’s T test and analysis of variance and linear regression analysis for comparison of slopes and intercepts. Survival comparisons were assessed by the Gehan-Breslow-Wilcoxon test.

## Results

### Infection and treatment initiation

**Fig 1:**
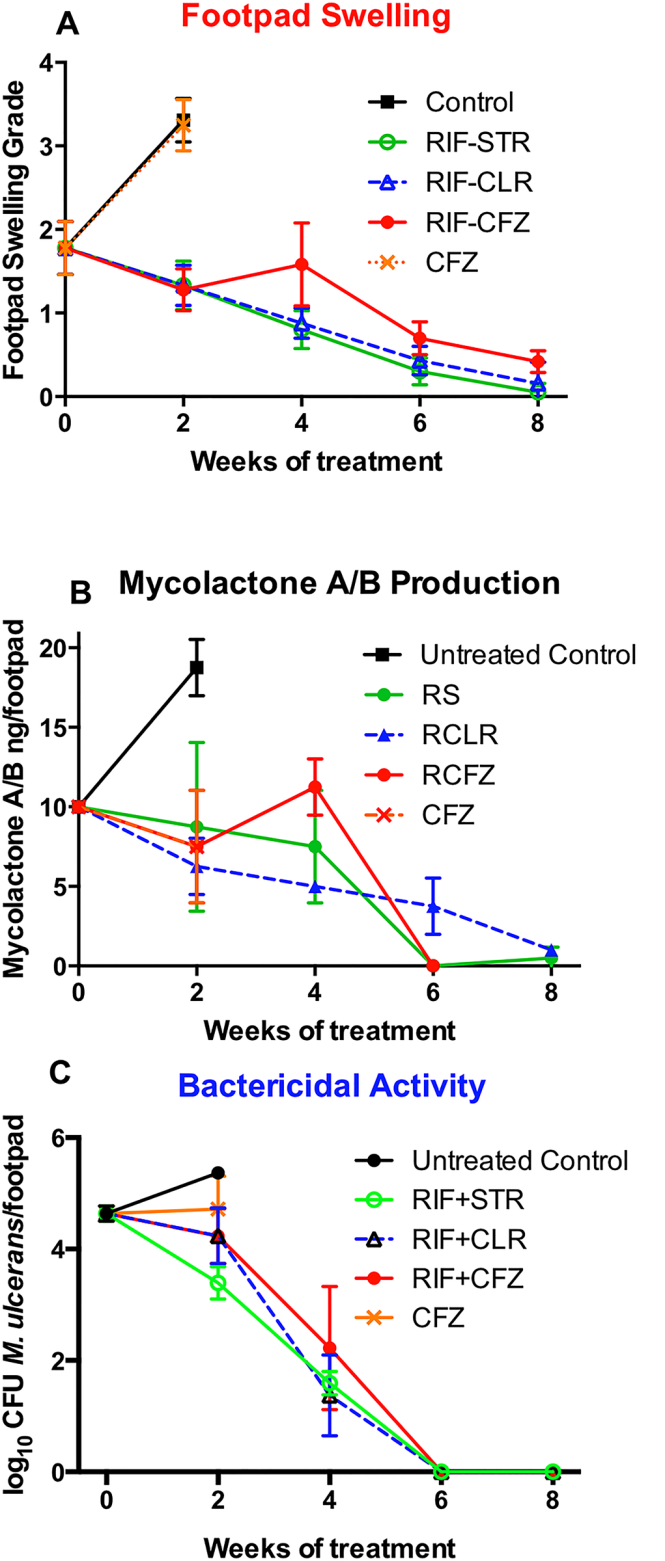
Response to antibiotic treatment in footpads of mice infected with *M*. *ulcerans*. **(A) Footpad swelling**. After infection of both hind footpads, treatment began when swelling approached grade 2 at week 7. Swelling continued to increase for the next two weeks in untreated control mice (black squares) and in mice treated with clofazimine (CFZ) alone, but was arrested in mice treated with either rifampin and streptomycin (RIF+STR, green circles), rifampin and clarithromycin (RIF+CLR, blue triangles) or rifampin and CFZ (RIF+CFZ, solid red circles) and then declined at a comparable rate to a grade of less than 1 over the 8-week treatment period. **(B). Mycolactone A/B concentrations**. At day 0, there was 10 ng mycolactone A/B per footpad detected. Mycolactone concentrations continued to increase for the next two weeks in untreated control mice (black squares), reaching 20 ng. Mycolactone production was arrested in mice treated with either RIF+STR (green circles), RIF+CLR (blue triangles), RIF+CFZ (solid red circles), or CFZ alone (orange X) and became undetectable after 6 weeks of RIF+STR or RIF+CFZ. The decline was slower in RIF+CLR mice. **(C) *M*. *ulcerans* CFU**. At day 0, there were 4.64±0.14 *M*. *ulcerans* CFU detected in the footpads. CFU numbers increased in untreated controls to 5.37±0.07 by day 10. The CFU levels remained essentially unchanged in mice receiving CFZ alone (4.72±0.59) while in the combination regimens, there was a consistent decline and all mice were culture negative by week 6.

Treatment was initiated seven weeks after the inoculation of 1.89±0.23 log_10_ CFU of *M*. *ulcerans* strain Mu1059, when the mean footpad swelling index was 1.78±0.32 [29], the mean CFU count was 4.64 ± 0.14 log_10_, mycolactone A/B concentrations averaged 10 ng per footpad (Fig 1).

### Response to treatment

Mice were evaluated daily. Failure to respond to treatment, as manifested by progressive footpad swelling and ulceration, necessitated euthanasia of some mice. Untreated mice and mice treated with CFZ alone were euthanized at day 12, after only 9 doses of CFZ due to deteriorating footpad lesions. At this time the swelling grade had advanced to 3.31 ± 0.26 and 3.25 ± 0.31 in the control and CFZ alone mice, respectively (Fig 1A). Mycolactone A/B concentrations were higher among untreated controls compared to CFZ-treated mice (18.75 ± 1.77 ng/100 mg footpad versus 7.50 ± 2.39 ng/footpad, respectively) (Fig 1B). However, due to co-migration of a pink spot with mycolactone in the TLC, the concentrations may not have been accurately discerned in CFZ-treated mice. Mean CFU counts in footpads were 5.37 ± 0.07 and 4.72 ± 0.59 in control and CFZ alone mice, respectively (Fig 1C). From these data, we conclude that treatment with CFZ alone had little or no impact on lesion size and bacterial load. At the initiation of treatment, swelling grade, an indicator of disease severity, was similar in all the groups except that the RIF+STR group had a lower swelling grade than other groups (S1A Fig). By day 11 after treatment initiation, 9 RIF+STR, 16 RIF+CLR, and 37 RIF+CFZ mice had been euthanized from among the 60 mice allocated to each group. In terms of survival, treatment with RIF+CFZ was superior to both no treatment (p<0.0001) and treatment with CFZ alone (p = 0.0023) but inferior to RIF+STR and RIF+CLR (p <0.0001 and p = 0.002, respectively) (S1B Fig).

Among surviving mice, the combination regimens resulted in improvement in all parameters. By week 2, footpad swelling indices decreased to 1.33 ± 0.29, 1.33 ± 0.24, and 1.28 ± 0.25 in mice treated with RIF+STR, RIF+CLR, and RIF+CFZ, respectively (Fig 1A). Mycolactone A/B concentrations declined to 8.75 ± 5.30, 6.25 ± 1.77, and 7.50 ± 3.54 in these same groups, with the same caveat about estimating mycolactone concentrations in CFZ-treated mice (Fig 1B). CFU counts were lowest among mice treated with RIF+STR (3.39 ± 0.29), but were also lower among mice treated with RIF+CLR (4.24 ± 0.50) and RIF+CFZ (4.23 ± 0.49) (Fig 1C). By week 4, footpad swelling grades averaged less than 1 in RIF+STR (0.8 ± 0.23) and RIF+CLR (0.88 ± 0.18) treated mice but were not significantly reduced in RIF+CFZ (1.58 ± 0.50) treated mice. Mycolactone A/B was detectable at 7.50 ± 3.54, 5.00 ± 0.00, and 11.25 ± 1.77 ng/footpad in the three groups, respectively. CFU counts declined to 1.59 ± 0.21, 1.37 ± 0.73, and 2.22 ± 1.11 in the RIF+STR, RIF+CLR, and RIF+CFZ groups, respectively. At week 6, mean footpad swelling was limited, with mean grades of 0.30 ± 0.16, 0.43 ± 0.17, and 0.70 ± 0.20. Mycolactone A/B was undetectable in the RIF+STR and RIF+CFZ mice while small amounts (3.75 ± 1.77 ng/footpad) remained in the RIF+CLR mouse footpads. CFU counts were uniformly negative at this time point. By week 8, swelling had disappeared in most footpads of RIF+STR treated mice (0.05 ± 0.11), was minimal in RIF+CLR treated mice (0.16 ± 0.26) and was limited in RIF+CFZ treated mice (0.42 ± 0.13). Traces of mycolactone A/B were detectable in RIF+STR mice (0.50±0.71) and RIF+CLR mice (1.00±0.00); RIF+CFZ mice were not tested at this time point in order to reserve mice for week 8 CFU and week 6-relapse analysis. No CFUs were isolated from the footpads of mice treated with all combination regimens. We conclude that RIF+STR has the most rapid bactericidal effects while RIF+CLR and RIF+CFZ, with time, were ultimately as effective as RIF+STR in arresting footpad swelling and eliminating cultivable bacteria.

### Clofazimine accumulation in mouse footpads

**Fig 2:**
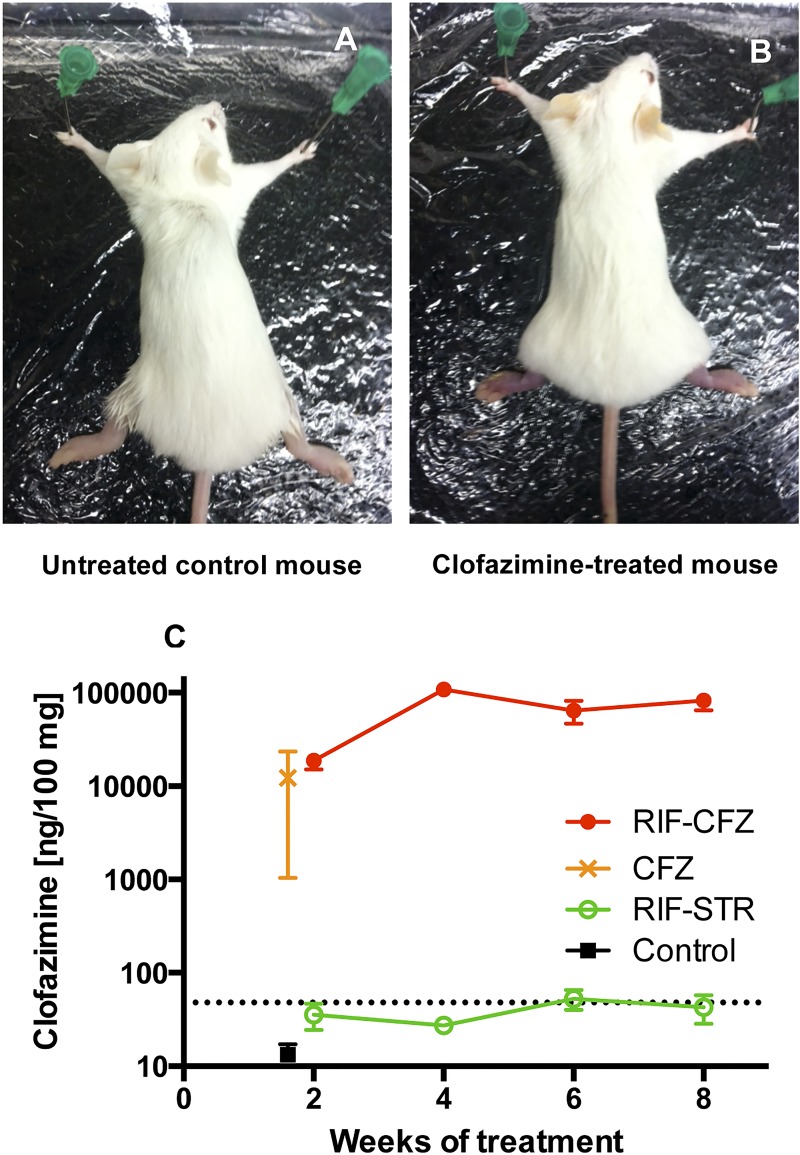
Clofazimine accumulation in tissues of *M*. *ulcerans* infected mice. (A) Untreated control mouse, showing normal skin (i.e. ears, tail, footpads) coloration. (B) Mouse treated with CFZ, 11 days after treatment initiation; note yellow-orange tinge to ears, dark red footpads and tail. CFZ-related discoloration was no longer apparent 3 weeks after the cessation of treatment. (C) CFZ concentrations detected by mass spectrometric analysis in CFZ mice (orange X) and untreated control mice (black square) at day 11 and RIF+CFZ mice (solid red circles) and RIF+STR mice (green circles) at weeks 2, 4, 6, and 8 of treatment. Dotted line, lower limit of detection.

CFZ is well known to cause discoloration of tissues, particularly ears and tails of mice and subcutaneous fat, and organs such as lung and spleen [17,18]. Compared to control mice (Fig 2A), we noted both increased inflammation and reddening of footpads in mice treated with CFZ (Fig 2B, S2 Fig) whereas other mice treated with, e.g., RIF+CLR (S2 Fig) had inflammation of footpads but no discoloration of ears or tails. To confirm that CFZ reached infected footpads, we carried out a pharmacokinetic analysis. Mice treated with CFZ alone were found to have 6.97 ± 4.12 μg/100 mg footpad tissue 11 days after treatment initiation at which time all mice in this group were sacrificed. Mice treated for 2 weeks with the combination of RIF+CFZ had 14.88 ± 5.30 μg/100 mg footpad and the concentration increased to 93.85 ± 23.14 at week 4 before plateauing at 55.20 ± 14.26 and 56.10 ± 11.32 μg/100 mg footpad at weeks 6 and 8. The discoloration waned and was no longer detectable three weeks after treatment cessation. Untreated and RIF+STR controls had no detectable CFZ (Fig 2C).

### Relapse after treatment cessation

Mice were monitored weekly for recurrence of footpad swelling after treatment cessation. Footpads in which a recurrence of swelling was observed were taken for histologic and microbiological confirmation of the presence of *M*. *ulcerans*.

**Fig 3:**
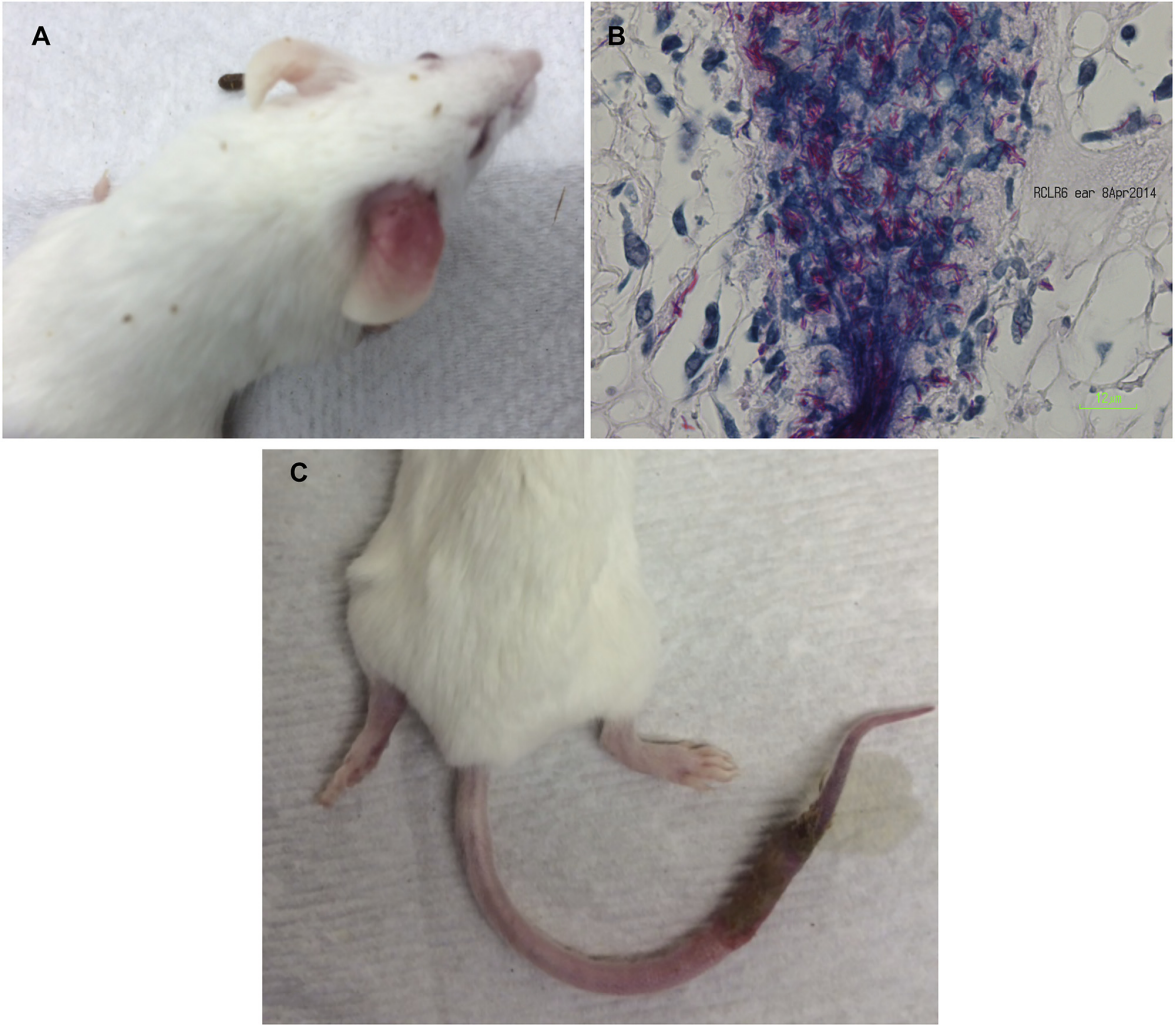
Relapse after treatment completion. Mice were monitored weekly for renewed swelling of infected footpads after treatment completion. In four mice, lesions instead appeared at ectopic sites (A) Mouse treated with RIF+CLR for 6 weeks began to show inflammation at the base of the right ear ~14–15 weeks after treatment cessation and was sacrificed at week 21. (B) AFB staining of lesion in right ear shows abundant bacilli as did growth of bacilli in culture at 32°C. (C) Mouse treated with RIF+CLR for 8 weeks showed essentially no sign of relapse and no cultivable bacilli in footpads but had a severe lesion at the base of the tail 31 weeks after treatment completion with abundant AFB and cultivable bacilli.

**Fig 4:**
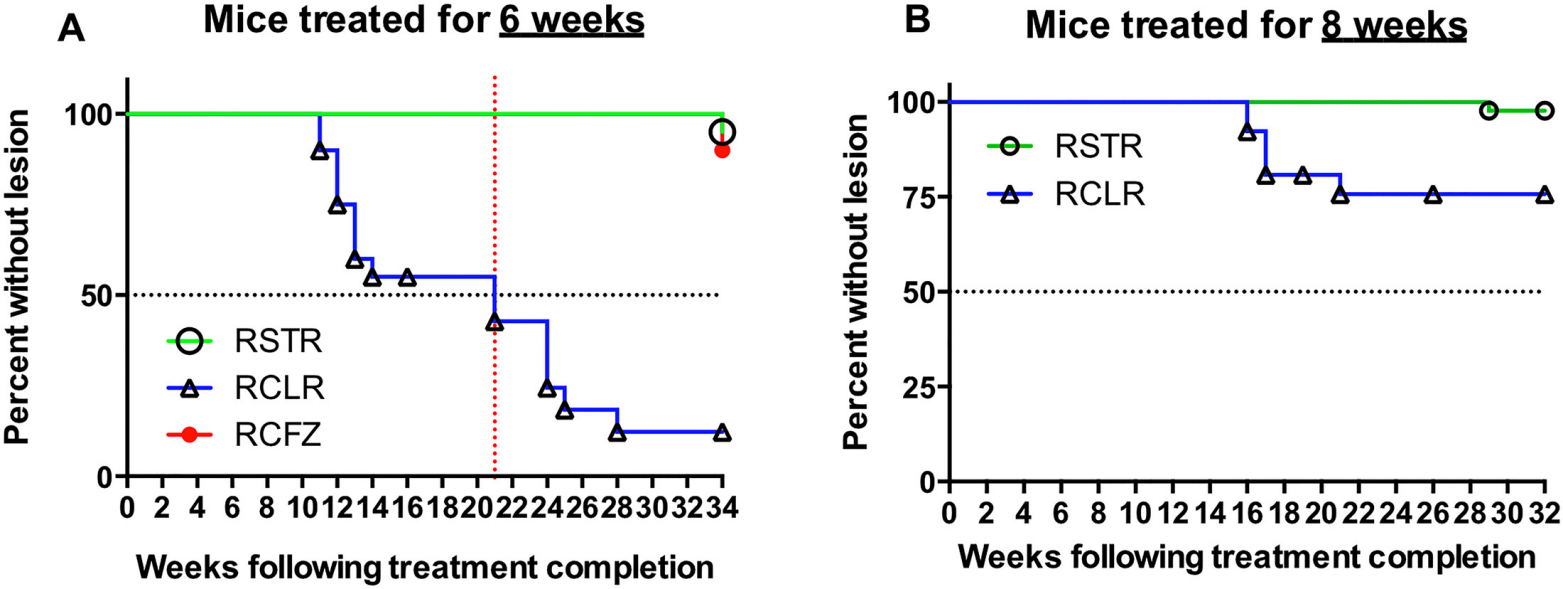
Time to footpad swelling after completion of antibiotic treatment. Time to footpad swelling (or ectopic lesions) in mice treated with either RIF+STR (green circles), RIF+CLR (blue triangles), or RIF+CFZ (red circles) regimens for six weeks (A) or eight (B) weeks after the development of swollen foot pads. Footpads had little or no swelling at treatment completion (see Fig 1A).

Among mice treated for 6 weeks, all (10 mice, i.e., 20 footpads for RIF+STR and RIF+CLR groups; 5 mice, i.e., 10 footpads for RIF+CFZ group) treated with one of the combination regimens remained relapse free for 10 weeks after treatment. After 10 weeks, mice treated with RIF+CLR started to display swollen footpads that progressed from grade 1 to grade 2. By week 28 following treatment cessation, 50% of RIF+CLR-treated mice had recurrent footpad swelling. Additional mice treated with RIF+CLR for 6 weeks developed lesions after treatment cessation, resulting from dissemination beyond the footpads—on the ear (Fig 3A and 3B) or tail (Fig 3C) without showing relapse in the footpad. The median time to relapse as assessed by development of a lesion anywhere in the body was 21 weeks in this group (Fig 4A). In contrast, mice treated with RIF+STR or RIF+CFZ for 6 weeks remained relapse free with the exception of a single RIF+CFZ mouse in which dissemination to the tail was observed at week 33 just before the end of the experiment at week 34. The presence of *M*. *ulcerans* was confirmed in all footpads by the presence of numerous acid-fast bacilli at histology and/or culture.

Among mice treated for 8 weeks, all (21 mice, i.e., 42 footpads for RIF+STR and 13 mice, i.e., 26 footpads for RIF+CLR groups) treated with one of the combination regimens remained relapse free for 15 weeks after treatment at which time, mice treated with RIF+CLR began to show relapse. However, the percentage of recurrent swelling of footpads after 8 weeks of treatment did not exceed 25% (Fig 4B). Treatment with RIF+STR for 8 weeks prevented relapse in all mice.

## Discussion

Although the initial bactericidal activity of RIF+CFZ was worse than that of RIF+STR and RIF+CLR (as measured by CFU and the number of mice requiring euthanasia), the sterilizing (relapse protection) was comparable to RIF+STR and superior to the currently proposed all-oral regimen of RIF+CLR. The delayed bactericidal activity of CFZ-containing regimens against *M*. *ulcerans*, associated with increased sterilizing activity (absence of relapse), parallel what has been observed against *M*. *tuberculosis* [17,18]. The consequences of relapse are potentially much more serious in tuberculosis, a fatal disease, than in Buruli ulcer, a disabling disease. However, adequate treatment durations are required to prevent further morbidity and the need for retreatment in either disease. Limited data indicate that relapse is rare in patients treated with RIF+STR whereas paradoxical reactions that may be confused with relapse are more frequent [32,33]. Given the anti-inflammatory properties of CFZ, its incorporation into BU therapy could potentially reduce the frequency of paradoxical reactions in addition to reducing the treatment duration necessary for cure.

The superior sterilizing activity of RIF+CFZ compared to RIF+CLR suggests that the former may be a more promising all-oral regimen to eliminate or minimize the need for STR, an injectable drug that also has potential toxic side effects. However, despite the drawbacks of STR, its potent antibacterial activity should not be overlooked. These tradeoffs have shaped the design of recent clinical trials that have adopted the concept of intensive and continuation phase regimens that are standard in the treatment of TB. In these studies treatment with RIF+STR was reduced from the WHO-recommended 8 weeks to 4 and even 2 weeks with a continuation of treatment with RIF+CLR for the balance of the 8 weeks [10,11]. The results were favorable with all of these alternatives and limited exposure to STR but did not totally eliminate it. At this time, the frequency of relapse after RIF+CLR treatment is not known. Interestingly, there are reports [5,9] of cases of nodular, non-ulcerating Buruli ulcer not responding to this regimen.

The principal drawback of CFZ is the skin discoloration that results from its administration. However the negative cosmetic impact of such skin discoloration is likely to be minimal, and to resolve rapidly, with treatment durations of 8 weeks or less.

Although the initial response to CFZ treatment was delayed, as is also seen in TB [17,18,34], mean footpad swelling and detectable mycolactone A/B in any combination regimen was reduced comparably after two weeks of treatment in the remaining mice while the CFU counts (bacillary burden) were significantly (p = 0.01) lower in the RIF+STR mice than in the RIF+CLR or RIF+CFZ treated mice.

As in other mycobacterial diseases, it is important to have a balance of early bactericidal activity, sterilizing activity, safety, and administrative simplicity in a drug regimen. *M*. *ulcerans* disease requires antibiotic treatment for only 8 weeks—much shorter than the times for tuberculosis (6 months for drug-susceptible TB) or leprosy (6 months for paucibacillary leprosy and at least one year for multibacillary leprosy)—even though the bacterial burden per gram of tissue can be extremely high in *M*. *ulcerans* lesions. Achieving a better balance in the treatment of *M*. *ulcerans* disease therefore might include treatment for one or two weeks with three drugs: RIF, STR, and CFZ. Beyond the initial phase, the duration of which remains to be determined, STR could be discontinued having achieved the early bactericidal activity. RIF and CFZ could be continued to avoid STR-induced toxicities, provide continued bactericidal activity, allow for outpatient drug administration, and provide strong sterilizing activity. CFZ is administered for two years normally for lepromatous leprosy with transient skin discoloration being the only side effect. This proposed regimen of three drugs for the first one or two weeks followed by RIF+CFZ alone could potentially also be shortened to 6 weeks or less with no loss of therapeutic benefit. Future experiments will test these regimens in the mouse model of *M*. *ulcerans* disease.

Our study has several potential limitations. The first is that the greater early mortality observed in the RIF+CFZ group may have introduced a survival bias that exaggerated the sterilizing activity of this combination relative to the other regimens. Nevertheless, the fact that only 2 (of 20) footpads of mice treated with RIF+CLR survived without relapse more than 28 weeks after treatment compared with all 10 footpads of mice treated with RIF+CFZ for the same duration indicates superior sterilizing activity of the RIF+CFZ regimen even if approximately twice as many RIF+CFZ-treated mice could not be assessed due to early mortality.

The second potential limitation is that relapse in mice cannot be equated with relapse in human BU patients. Evidence that BU may heal spontaneously and that some lesions harboring viable bacteria at the end of treatment resolve without further treatment suggest that the human immune response participates in the sterilization process, perhaps more than the murine immune response does [10,11,35]. However, even if one assumes that sterilization of BU lesions by chemotherapy is not necessary for cure in some patients, the duration of therapy needed for efficacy in a population is likely to be determined by those patients with an inadequate host response, where chemotherapy may have a more important role in sterilization and ultimately determine the duration of treatment needed for the population as a whole. We consider relapse to be the best outcome measure for comparing the relative ability of regimens to eradicate persisting bacteria in mice and use it under the assumption that the duration of treatment required for efficacy in the clinical setting is determined by the rate at which such persisting bacilli are eliminated. In this context, RIF+CFZ had superior sterilizing activity over RIF+CLR. Only future clinical trials comparing RIF+CLR and RIF+CFZ head-to-head as part of a treatment regime will enable further evaluation of the predictive accuracy of this experimental endpoint.

## Supporting Information

S1 Fig Survival of mice by treatment group. (A). Distribution of swelling grades at the initiation of treatment. Swelling was similar at baseline in the RIF+CLR and RIF+CFZ groups as well as the CFZ only group but markedly less in the RIF+STR positive control group. (B) Antibiotic treatment prevented euthanasia in mice with footpad lesions resulting from infection with *M*. *ulcerans*. Survival was significantly higher during the first two weeks of treatment in mice receiving RIF+STR or RIF+CLR compared to RIF+CFZ.
(TIFF)

S2 Fig Skin discoloration in mice receiving CFZ. Skin color is normal in mouse treated with RIF+CLR for 6 weeks (left) whereas in mice treated with RIF+CFZ for 4 weeks (right) the ears have a yellow-orange tinge and tail and footpads are dark red. The discoloration was no longer apparent 3 weeks after the cessation of drug treatment.
(TIF)

S1 Dataset CFU, Mycolactone A/B, Footpad swelling records, and CFZ levels. (XML)
