# Supplementary figures and images for: Efficacy of Rifampin Plus Clofazimine in a Murine Model of Mycobacterium ulcerans Disease
Source: PLoS Negl Trop Dis. 2015 Jun 4;9(6):e0003823. doi: 10.1371/journal.pntd.0003823 (PMC4714850; doi:10.1371/journal.pntd.0003823)

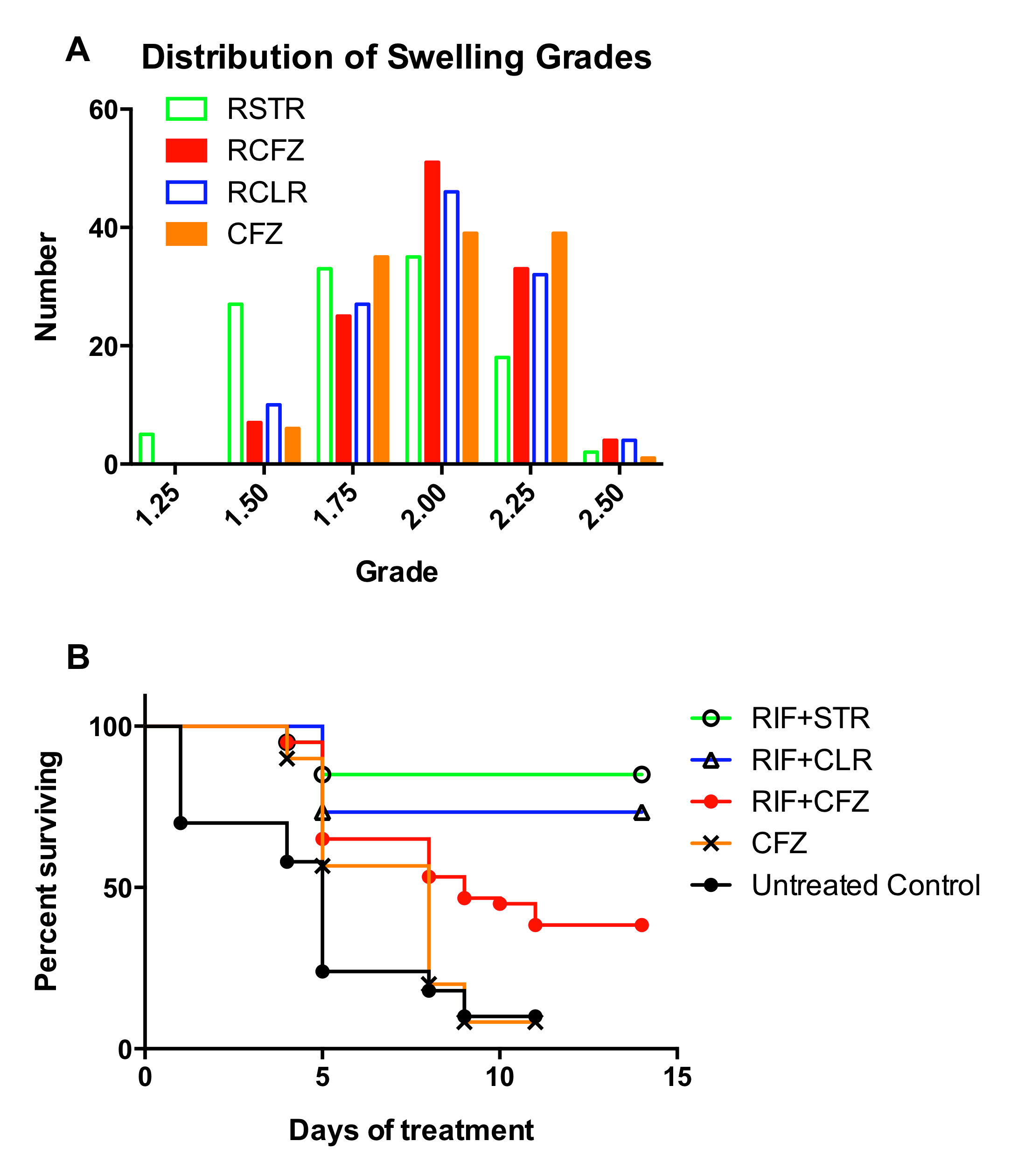

Supplement: S1 Fig — (A). Distribution of swelling grades at the initiation of treatment. Swelling was similar at baseline in the RIF+CLR and RIF+CFZ groups as well as the CFZ only group but markedly less in the RIF+STR positive control group. (B) Antibiotic treatment prevented euthanasia in mice with footpad lesions resulting from infection with M. ulcerans. Survival was significantly higher during the first two weeks of treatment in mice receiving RIF+STR or RIF+CLR compared to RIF+CFZ. (TIFF) [file pntd.0003823.s001.tiff]

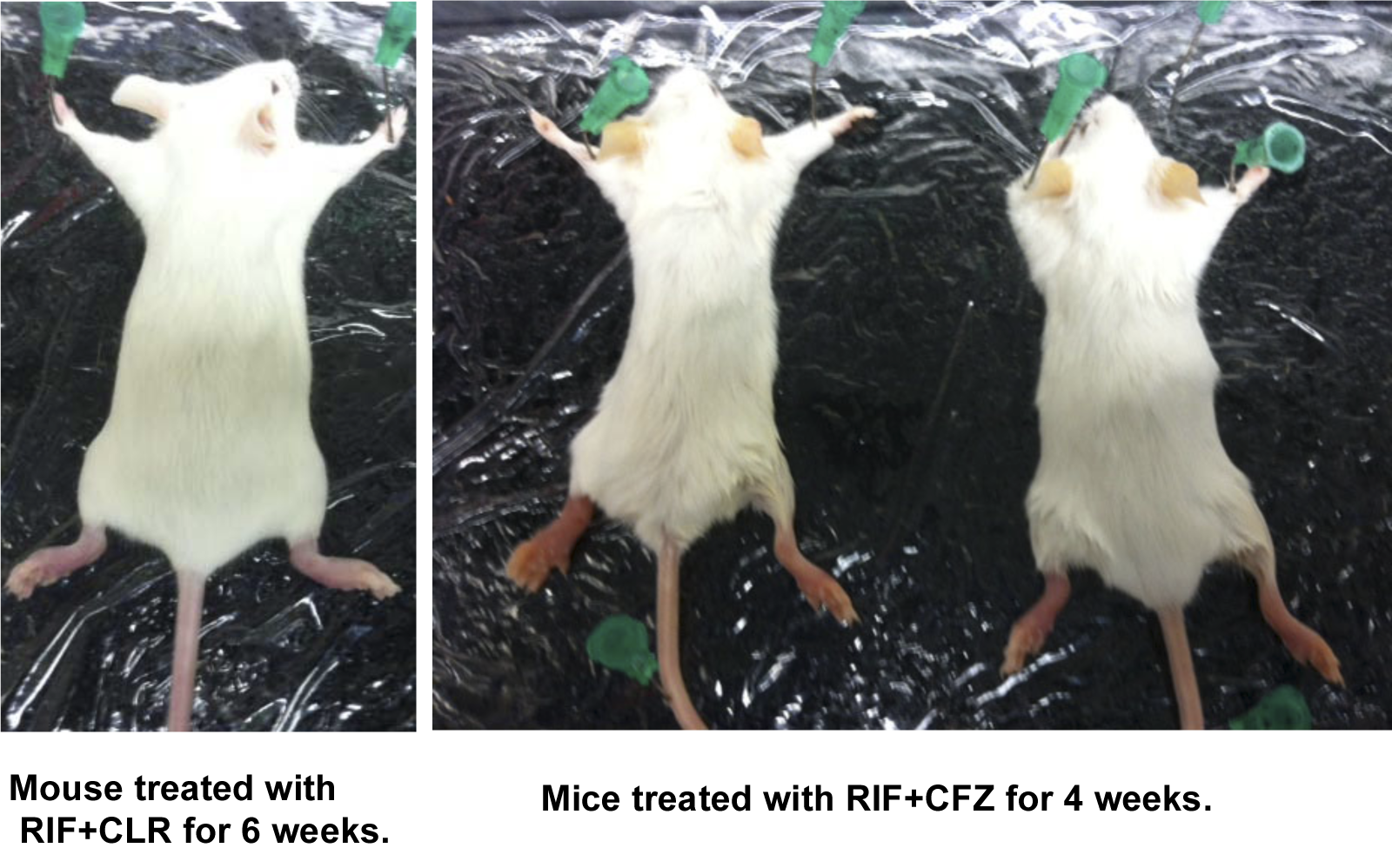

Supplement: S2 Fig — Skin color is normal in mouse treated with RIF+CLR for 6 weeks (left) whereas in mice treated with RIF+CFZ for 4 weeks (right) the ears have a yellow-orange tinge and tail and footpads are dark red. The discoloration was no longer apparent 3 weeks after the cessation of drug treatment. (TIF) [file pntd.0003823.s002.tif]
